# Social accessibility in queering code/space: Blued-based visibility and social practices of local gay people in mainland China

**DOI:** 10.3389/fpsyg.2024.1413725

**Published:** 2024-06-27

**Authors:** Tongwen Xu, Yansong Zhang

**Affiliations:** ^1^College of Literature and News Communication, Guangdong Ocean University, Zhanjiang, Guangdong, China; ^2^School of Journalism and Communication, Minzu University of China, Beijing, China

**Keywords:** Blued, mainland China, queering code/space, social accessibility practices, visibility negotiation

## Abstract

As a locative media, Blued articulates physical and digital space in the form of localization, giving a mediated visibility to its users who are not visible in physical space, thus making the city a queering code/space. This can transform social accessibility and the ways that gays come together and interact in local spaces. Taking mainland China, dominated by heteronomativity, as a field, this paper focuses on the visibility negotiation and social accessibility practices in Blued-based queering code/space. Using the digital anthropology method, this study found that Blued-based visibility is essential for non-closeted gay men to gain a sense of local belonging and community inclusion. Offline accessibility is a crucial demand for Blued users, but until then they have negotiated visibility through selective disclosure of social cues. At the same time, Blued users face visibility asymmetries and risks in the pursuit of accessibility. This study then argues that in the Blued-based queering code/space, the gay men constantly reconcile accessibility with others by managing visibility in a heteronormative urban space. In addition, this paper highlights the value of the hybrid of new media and physical space for the public interaction of marginalized groups in the city.

## Introduction: from Dianr(点儿) to Blued-based queering cities

In China's Confucian cultural tradition, marriage, children, and family are considered essential for maintaining family lineage and heritage. Within this cultural context, homosexuality is viewed as conflicting with traditional family values, making it difficult to gain social acceptance. Additionally, the current legal system in China lacks clear regulations regarding the legal status of homosexuality. Furthermore, while increasing numbers of people have started to accept and understand homosexual individuals in recent years, this acceptance still clashes with mainstream values, and widespread societal prejudice against homosexuality persists. Chinese society remains predominantly heteronormative (Luo et al., 2022). In this environment, physical spaces, which are crucial for social interactions, remain inhospitable to homosexual individuals, preventing them from freely engaging in public areas dominated by heteronormative norms. However, the advancement of digital communication technologies has transformed the spaces where gay men connect.

Since the reform and opening-up, the Chinese public homosexual space has evolved. Before the 1990s, “traditional public space” was representative and mainly concentrated in city center parks (often with public toilets and green spaces), squares, tea gardens, bathrooms, and dance halls for the public. After the 1990s, homosexual commercial public spaces began to flourish in bars, saunas, clubs, etc. On the one hand, traditional public space “has a low entry threshold” and “can be used with the majority of heterosexual people,” meaning that identifying a member of the group can only be achieved through eye contact, actions, or conversation.^1^ On the other hand, commercial public space “requires a fee to enter” and “is an exclusive space for homosexuality” (i.e., one does not have to make judgments about homosexuality in such spaces) (Wei, 2015, p. 23–24). These “public spaces where people in the circle communicate and entertain are called *Dianr(*点儿*)*,” in which “the physical, behavioral, and social practices of homosexuality are organized, expressed, and understood” (Fu, 2012, p. 55). Leap (1999) refers to it as “public sex/gay space.” Some scholars refer to it as “gay cruising spaces in China” (Qian, 2014). In China today, heterosexuality is considered a normal sexual orientation, while homosexuality is abnormal and rejected by mainstream values. The majority of gay men have not come out publicly. In this context, *Dianr* has become an essential space for gay men to avoid the risk of sexual orientation disclosure in heterosexual spaces and to interact with gay men.

Before the rise of the Internet, the urban gay community mainly used these Dianr to connect with other strange gay men in the local area (Beck, 1997, p. 105–106). In the Internet era, Douban, QQ, Tieba, etc.,^2^ provide platforms for gay groups to carry out social interactions. Individuals form a community imaginary in cyberspace, but there is no distinct physical space to point to. Since offline, physically present interactions are still irreplaceable for this group, people can only make up for the de-territorialization of such social media by using hometown dating groups (e.g., X-city gay friend groups in Tieba, Douban, QQ).

An increasing number of social media platforms now incorporate geographic location information (Fitzpatrick et al., 2016). The combination of location and social media has given rise to a new form of media, specifically location-based social networking (LBSN). LBSN detects the user's location through a GPS sensor and associates social activity with the location. In LBSN, people not only communicate about location but also communicate through location (Frith, 2014). As a kind of LBSN, with “nearby” as its outstanding feature, Blued is the world's largest gay social app (Miao and Chan, 2020), allowing users to see a list of other users based on their geographic distance. The list may be displayed in grid or list modes. Information that users can access includes profile image, name, whether the user is verified (marked with a “V”), distance, age, height, weight, signature, etc. Users can filter and search for other users of their choice according to their needs, and can also be “seen” by more people through the setting of related filter information. Blued acts as a filter to exclude heterosexuality from the physical space, and this filtering function was previously achieved through *Dianr* (this is not to say that the “Dianr” in the city have been completely replaced by Blued, but that gay men are now able to socialize with gay men in the same city without having to go to the “Dianr”). Therefore gay men with social needs have certain visibility on Blued, and the urban space is queered in this digital form and becomes a queering space.

Modern Chinese society is dominated by “heterosexuality” (Warner, 1993, p. xxi). Many cities' institutional arrangements are produced under this framework, and the sexual and social rights of the gay community are excluded. “Public space is ‘queering' by the raised visibility of the gay community, thereby they can enjoy the same citizenship as heterosexuals,” which is “an important part of contemporary gay space politics.” Furthermore, the city's public space “is supposed to be an area where people can use it equally, but in fact, many people are excluded from the public space.” The homosexual community is one of them (Wei, 2011). In this sense, Blued's ability to make public space queering is significant for the gay community in mainland China to engage in public interaction and gain social accessibility to the gay community.

On Blued, the importance of physical space and its articulation with cyberspace are unprecedentedly prevalent. However, little existing research on Blued has been conducted in this respect. The available studies have been done in the following: the political economy of sexually affective data on Blued (Wang, 2019, 2020); how sociocultural context matters in self-presentation on Blued (Chan, 2016); communication skills on Blued (Wu and Ward, 2020); how the different life experiences of gay men shape and are shaped by the use of these applications (Miao and Chan, 2020). However the hybrid of physical and digital spaces, and the implications of this convergence for changes in urban space and the social interactions of the gay community, have not been discussed in the above studies.

This paper discusses the social interaction of gay individuals on Blued from the perspective of media and communication geography. From the perspective of spatial convergence, social accessibility, and visibility, this paper examines what features of Blued-based local communities in mainland China are, what kind of interaction space they create for gay men, and what kind of interaction practices they promote, and then asks what they mean for gay men. This study argues that Blued, as a location-based media platform, merges physical and digital spaces in a localized form, providing a mediated visibility for Blued users who are otherwise invisible in physical space. It effectively reshapes the urban environment into a space imbued with queer identity. As a result, it transforms the spatial structure of social accessibility for gay male communities within the city.

## Literature review

### Social accessibility: the concept and its spatio-temporal structure

The concept of social accessibility can be traced back to Simmel (1922 [1955]) with its view that it is a way of managing interpersonal relationships in a city of sensory overload. In the opinions of Quan-Haase and Collins (2008); Pieber (2021), social accessibility can be understood as an ability to reach others. In terms of reaching and being reached, social accessibility includes two aspects: on the one hand, the ability to reach the people one wants to interact with; and on the other hand, the ability to manage who, where, when, and how one can interact with oneself. From existing research, the latter has been discussed more often. For example, in the view of Milgram (1970), urban life is characterized by “large numbers, density, and heterogeneity.” Milgram (1970) calls this phenomenon of overload caused by too much input for people to cope with, and argues that “adaptations to urban overload create characteristic qualities of city life that can be measured.” According to Milgram's (1970) observations, ways to cope with this overload include “allocation of less time to each input,” “disregard of low-priority inputs,” “boundaries are redrawn in certain social transactions” and so on.

The development of information technology has also changed the social accessibility of urban people. This social accessibility has become more complex and diverse in the mobile digital age (Quan-Haase and Collins, 2008). Social visibility is managed in a variety of ways, with time being an important dimension. Zerubavel (1979) outlines a conceptual framework of social accessibility and distinguishes between private time, where individuals are legitimately inaccessible to others, and public time, where they can be reached. Quan-Haase and Collins (2008) argue that the temporal structure of social accessibility and individuals' definitions of public and private time has changed in computer-mediated communication.

In addition to time, space is also an important dimension for discussing social accessibility (Gehl, 1987). Mobile internet development has also changed the way people interact in public spaces (Humphreys, 2010). According to Carr et al. (1992, p. 45), “public places afford casual encounters in the course of daily life that can bind people together and give their lives meaning and power.” The provision of public space is an important social feature of a city, in which people can interact, scrutinize, communicate, and debate with one another (Calhoun, 1986). Digital space was once considered dichotomous with physical space, but this view has since been abandoned. In Mitchell's *City of Bits*, for example, it is clear that “bits” will replace “bricks” as the dominant city (Mitchell, 1996). However, in *Placing Words*, he abandoned this dichotomy between digital and physics, arguing that the two are intertwined (Mitchell, 2005). This convergence redefines urban space and also influences the management of social accessibility for urban dwellers.

In summary, social accessibility encompasses the ability to reach others and manage others' access to oneself, and it is mainly discussed in the context of urban overload. For marginalized groups, however, the lack of communication between members scattered across the city in invisible states is often due to a lack of information. The articulation of digital and physical spaces creates the conditions for accessibility among urban minorities.

### Queering code/space: Blued as locative media and hybrid space

Locative media is a digital mobile media with a location-aware function, which senses a user's specific location in physical space with positioning equipment, providing relevant information nearby (Oppegaard and Grigar, 2013; Frith, 2015, p. 2). In locative media, the significance of physical space for network communication has been emphasized. What information users learn is closely related to where these users are (Gordon and de Souza e Silva, 2011, p. 7). Locative media is a manifestation of the expansion of networked digital media in urban space, representing a “new spatialization of media as an integral part of the transformation of media into geomedia” (McQuire, 2016, p. 2). It shows the articulation between the media and urban space, changing people's ability to experience space. To highlight the interweaving of the two spaces in this mobile digital era, de Souza e Silva (2006) proposes the concept of “hybrid spaces,” which emphasizes the fusion and interaction between cyberspace and physical space rather than a binary opposition. Silva (2009) believes that, in hybrid spaces, mobile technology serves as an interface, blurring traditional boundaries between physical and digital space, thereby reconstructing urban space. A continuous relationship is established among locative media, users, cyberspace, and physical space in the hybrid process. This relationship promotes a new form of space and redefines interpersonal and social relations within the space (Saker and Frith, 2019).

For the gay community, communication is increasingly mediated by mobile applications (Mowlabocus, 2008, 2010; Race, 2015). For example, Grindr, one of the most popular gay men geosocial applications in Western countries, Roth (2014), Bonner-Thompson (2017), and Miles (2017) believe that it broke the boundary between public and private space and provided a new space for queer's gender, sexual behavior, identity construction, embodiment, and performance. Following Kitchin and Dodgem's (2011) concept of “code/space,” Cockayne and Richardson (2017) refer to the space created by the hybrid of gender, digital technology, and geography as “queering code/space.” To Blued, it filters heterosexuality in physical space through networked and localization forms, and gay men in physical space can perceive other gay men in their surroundings digitally, thus making the city a queering space. In the process, Blued creates a digital layer on top of the physical world, allowing for new social interaction practices (Mark and Marcus, 2012) and providing new complications to the issue of social accessibility in urban spaces (Pieber, 2021).

### Visibility negotiation and social accessibility practices in the queering code/space

Communication in social media is viewed as de-spatial. Regarding Blued, however, space is brought back into view in a locative way and is viewed as a self-disclosure of information by the user in the socialization process. The spatialization of self (Schwartz and Halegoua, 2015) gives rise to a new kind of visibility. Visibility can be divided into “visibility of body presence,” which is predicated on the perception of physical presence, and “mediated visibility,” which is realized in the form of mediation. Thompson (1995, p. 119–148, 2005) argues that “mediated visibility” extends the spatial scope of individual visibility, characterized by non-presence, non-dialogue, unlimited openness, etc. Treem et al. (2020) find that this “communication visibility is the root affordance, or possibility for action for CMC.” In Brighenti's (2012) view, visibility is associated with vision but beyond vision in that it “essentially regards the activity of introducing, establishing and negotiating thresholds that join together or separate territories” Territory is a concept closely related to visibility, which considers the change of threshold of visibility as an important motivation for domain formation (Brighenti, 2007). In this sense, the visibility brought by Blued also facilitates the process of re-territorialization of urban space. The visibility of Blued can also be seen as an act of re-territorialization of the cities. Pieber (2021) refers to locative media as a spatial filtering function, arguing that it affects visibility and social interactions of LGBTQ+ people.

However, this social accessibility should be seen as a concept of practice, emphasizing the intertwining of technology and people, nonhuman and human (Lupton, 2016) Specifically, in the field of media practice, it allows us to see media at work in many contexts and situations and understand how media practices arrange, combine, and more generally intersect with other social practices (Couldry, 2004). Mattoni and Treré (2014) argues that when following this concept of media practice, we should focus on the user interactions with media objects (such as mobile phones, laptops, pieces of paper) and media subjects (such as journalists, public relations managers, other media practitioners who are connected to the media realm). We can therefore divide this social accessibility practice into two aspects: on the one hand, we focus on the interaction between users and media technologies; on the other hand, we focus on the interaction between users and other people. An important manifestation of this social accessibility practice in the Blued-based queering code/space is that Blued provides “visibility affordances” (Flyverbom et al., 2016) for the gay community and the negotiation of visibility in the relevant cultural context.

In summary, Blued as a locative media has redefined urban space for its users, transforming the heteronormativity urban space into a queering code/space in which gay users are visible to each other, thus affecting the social accessibility of this group in the city. Nevertheless, what the significance of this visibility in queering code/space for the gay community in China is, how this visibility and social accessibility are negotiated in Chinese heteronormativity public spaces, and what social practices it engenders are the questions this paper seeks to explore next.

## Method

To conduct a rigorous investigation, we employed two methods for data collection. First, we used the walkthrough method developed by Light et al. (2018). This emerging digital experience method encourages researchers to conduct detailed examinations of applications, familiarizing themselves with the platform's interface design, functional structure, content, and specific practices. It involves systematically collecting data throughout various steps of the app (from registration to everyday use and discontinuation) to comprehensively understand the platform's ecological characteristics and how the platform system integrates with daily life practices.

The researchers in this project actively used Blued to familiarize themselves with the settings and functions of the relevant applications, gaining a perceptual understanding of these applications on a personal level. Starting from April 2, 2018, the two researchers first logged in and read the descriptions and promotions of the app in the app store and on Blued's official website. In this phase, we gained an initial understanding of its features and development history from official introductions. Subsequently, the two researchers installed Blued on their respective Android and Apple phones, used it for at least 30 min daily for a month, and collected extensive field notes, screenshots, and screen recordings.

During this participatory observation on the platform, aside from examining the basic functions and layout of the software, the researchers made additional finidings. For instance, many users proactively greeted the researchers, often starting the conversation with questions like “May I take a look at you?” “Do you have a photo?” “What's your situation?” These interactions were consistent with the experiences shared by the interviewees later in the study. Additionally, the researchers joined several chat groups within Blued and observed the interactions among gay men in these groups. The detailed process of using Blued was carefully documented, capturing both objective digital traces from smartphones and subjective user experiences as supplementary first-hand research materials.

In addition to collecting data through the walkthrough method, we conducted in-depth interviews, carried out from May 2018 to June 2019. Blued users are distributed across all cities in China, with the majority residing in first-tier cities such as Beijing, Shanghai, Guangdong, and Zhejiang.^3^ Given this context, we chose Shanghai (a first-tier city) and Zhanjiang (a third-tier city in Guangdong province) as the main locations for recruiting interviewees. We interviewed a total of 14 gay individuals (nine college students and five urban white-collar workers) through open recruitment and snowball sampling (see Appendix 1). The interviewees were mainly comprised of college students, and there were two reasons for choosing college students as the main object of observation. The first reason was the convenience of the method employed. Secondly, as a bounded area, universities are appropriate to discuss what kind of social and public interaction Blued has triggered. A further five urban white-collar workers were interviewed. Because these participants had the financial ability to enter places with consumption thresholds such as gay bars, clubs, and *Dianr*, the social environment around them is more complex than a campus, and their media experience complements that of the students. Although college students and urban white-collar workers do not represent all Blued users, data indicates that 70% of Blued users are between the ages of 18 and 35, with 30% aged 21–25 and 27% aged 26–35. These users primarily consist of college students and urban white-collar workers (see text footnote 3). Thus, this study concentrates on these two groups. Furthermore, this study aims to explore the spatial generation of Blued and its impact on social accessibility, rather than examining usage differences among various social strata and groups. Additionally, the primary objective is to investigate the spatial dynamics of Blued and its influence on social accessibility, without delving into the usage differences among various social classes and groups. Given the research goals, the chosen subjects are pertinent and meaningful for the study.

The interviews lasted an average of 35–45 min and followed a semi-structured protocol. At the beginning of each interview, the researchers asked participants to describe the homosexual dating apps they frequently use, providing a general overview and evaluation of these apps. Since all participants had experience using Blued, the researchers then focused specifically on this app. The main questions were as follows: when did you start using this app? What was your initial experience like? What significance does it hold for you? How do you find nearby users? How do you filter chat partners? What are your distance preferences and what do they mean to you? Are you concerned about being recognized by acquaintances on Blued? Have you ever been recognized? Do you usually visit physical spaces like gay bars? How do interactions in these physical spaces compare to those on Blued?

To ensure the validity of the experiential materials, researchers conducted participatory observation and in-depth interviews again from April to July 2023. We found that the interaction patterns among gay men in China have remained largely unchanged over the past 5 years. Blued continues to have a large user base and is still a popular social app among gay men. Apart from adding features similar to Douyin (TikTok) live streaming, the app's core functionalities have remained largely the same. In interviews conducted last year with two gay men about their use of Blued, the insights gained were similar to those from the study conducted 5 years ago.

The researchers then followed a grounded theory approach to deeply read and analyze the field materials. During the initial phase, the researchers employed open coding on the field materials, summarizing 11 key themes and concepts: “sense of belonging,” “finding a community,” “convenience,” “anonymity,” “meeting in person,” “online-to-offline transition,” “process dynamics,” “disguising,” “risk factors,” “inequity,” and “familiar strangers.” Following this, the researchers performed summarization and axial coding on these themes, categorizing and comparing them to derive three primary categories: the online community of local LGBTQ+ individuals, the procedural aspects of social accessibility, and the visibility risks within Queering Code/Space. These themes also serve as the core topics regarding visibility and social accessibility within Queering Code/Space facilitated by Blued, forming the basis for subsequent analysis.

## Findings

“I am grateful but conflicted. On the one hand, Blued makes us easier to find gay friends nearby anywhere and anytime. On the other hand, the whole process of socialization from online to offline seems like a ‘negotiation', and I might pay the price for it.”

H said the above when commenting on the significance of Blued to his daily life. His words represent both the value of Blued's availability to this group and the fact that this value is a negotiated process that needs to be considered in a specific cultural context. Based on fieldwork materials, the following findings emerge: firstly, in Blued-based queering code/space, gay individuals who cannot reveal their identities in physical space and chat with one another see each other in cyberspace. This visibility creates a user-centric, distance-oriented community within the region, filtering out the eyes of heterosexuals and becoming important for gay people, especially those who are not out of the closet, resistant to the *Dianr* or have unconditional access to the *Dianr*, to integrate into the local gay community. Secondly, in queering code/space, most users seek offline accessibility, such as meeting the netizens, dating, etc. Nevertheless, before that, they will negotiate visibility through selective disclosure of social cues. Finally, in the queering space based on *Blued*, there exists a paradox. On the one hand, Blued users hope to be searched by people nearby and establish a certain kind of relationship on this platform. However, on the other hand, some Blued users are afraid of being seen by acquaintances because of the actual relationship generated by the proximity of physical space. This asymmetry between seeing and being seen can be regarded as a paradox and risk of Blued-based visibility. The following sections discuss these aspects in detail using the specific experiences of participants.

### “A necessary threshold to enter the local gay community”: a Blued-based sense of place and queer community inclusion

The activities of Chinese homosexuals in a physical space dominated by heterosexual values are not recognized, either on an official or folk level. For example, at GMY University, in 2018, some gay individuals spontaneously established a “Rainbow Group” and created a WeChat official account. The group regularly held meetings outside the school. The reason for choosing off-campus meetings was that the use of on-campus space (such as classrooms, etc.) was subject to application, and the school could not approve this group. Their activities ranged from watching classic gay-themed movies together to talking about gay-related issues. However, when the school learned of the group's existence, it was ordered to disband, shut down its official accounts, and cancel movie-watching events. In addition to school authorities, gay individuals face pressure from traditional family values and social circles. On various social occasions, gay men such as B are reluctant to expose their sexual orientation to others. He said: “You should disguise yourself as straight to the largest extent possible; on the one hand, you don't know if the other person is gay. On the other hand, you also fear they'll tell someone else, which is embarrassing.”

Therefore, most gay individuals are in a state of a deep closet, meaning that they are invisible. Just as A states: “Our group wants to find the same kind of people, but we are afraid of being discovered by our classmates, friends, or other gay individuals.”

By filtering out heterosexuals and anonymization, Blued seems to have solved this paradox by allowing gay individuals to interact with other gay men without revealing their real identities. Later, according to the needs, the users will carry out de-anonymization in the interaction process.

In the past, in the same city, if gay individuals wanted to get to know one another, they may need to use one of the following methods: meetings arranged through friends; going to a gay bar; finding the city's QQ or Douban group, or private chat. However, there are some drawbacks for GMY students, as explained by E:

It is not realistic to strike up a conversation, even if you meet someone who looks gay on the street. You are unlikely to ask directly if they're gay and whether they like to friend you on WeChat. If you want to get a referral from a friend, you should have a friend or social circle. My friends are always in the same social circle as me; gay bars are impossible because none in my city, and my friends say they're messy. There are many kinds of people in QQ Gay groups in my city. I just want to find students, not people from society.

A is a senior at GMY University. He identifies as a bisexual and used Blued for the first time in his dormitory. He recalled the scene and feelings at that time:

I was shocked when I read how they spoke. One of the things that impressed me most was people on Blued were very close to me, some even at zero kilometers. I didn't know that there were so many gay youths in our school; when you scroll down, you find that there are many people within three kilometers.

Based on distance alone, A stated that fellow users belong to the university or even the same dormitory compound; a distance of fewer than three kilometers means that these people are likely to be students from GMY University.

C is a junior at the Dongguan University of Technology who has used Blued and Tantan since the second year of high school. C later also used Aloha, Forfun, Jackd, etc. When he first entered university, C said that he was shocked when he tried Blued in his dormitory for the first time: “Holy sh^*^t! There are so many gay youths in my dorm!”

Gay individuals get a new sense of place based on Blued. In general, a “sense of place” refers to people's subjective and emotional attachment to space, which is a feeling based on personal experience. This “nearby” function on Blued makes gay individuals visible. This kind of visibility, in addition to the traditional *Dianr*, allows for *Dianr* everywhere. This sense of place is important for gay men to enter their local social circle. As E stated: “Only with Blued did I enter the local circle. It's hard to get to know people without that. It's a kind of a barrier to entering the local gay community.”

Some people, including participants like D, E, and H, view Blued merely as an app for hooking up—designed primarily for finding sexual partners and engaging in sexual activities rather than for making friends or seeking emotional companionship—thereby making it a breeding ground for HIV. This notion, however, is a misunderstanding. When talking about the stigma of Blued, J said: “Blued is regarded as an app for sexual hook-up in most people's eyes, but everyone uses it for different purposes.”

This study found that social interactions on Blued were used for much more than meetings or sexual encounters. Blued users can also develop their relationships and build friendships with other people in the gay community, as stated by H: “It did help me with demand for sex. However, I made some friends there. I met friends who played volleyball with me through Blued and even some friends who are so nice that I can get along with them for years.” From the perspective of social network construction, this kind of friendship can enhance the social capital of both sides. As J stated: “We don't necessarily use Blued in that way (for sex); we also use it for friendships. I'm a student who stays on campus and see my classmates every day. Blued is a good way to enlarge my friend circle.” L, a doctorate student from Shanghai, also disagrees that Blued users are only for casual sex. L said he had recognized people who were “helpful” to him through the application. Helpful here refers to a person who can guide a user's life and study.

### Negotiation of visibility: accessibility as a process

Reproducing the value of physical space in online social interactions is a crucial feature of Blued. In localization, Blued users in the physical space have a certain degree of visibility online. Although some users, like E, use Blued for “chatting” and “killing time.” F stated the following:

One of the primary purposes for me to use Blued is to find people around me and have offline interactions with them rather than chatting online. If I only wanted small talk, I'd go for Aloha, Fangkaka, etc. On Blued, at some point, two people will ask to meet offline if they have a good conversation or are interested in each other online. If you feel good when meeting, you might want to continue the offline relationship. If you don't feel good, you might want to stay online or just stop talking.

Therefore, it may be defined that, in the Blued-based queering code/space, offline communication is always an “ultimate pursuit.” This purpose is also a key objective of choosing to chat with people nearby, highlighting the importance of physical presence in communication.

*Mian Ji* (meeting a net friend offline) is an internet buzzword in mainland China in recent years. The term *Ji* originally meant “bromance” or “homosexual.” Although the meaning of the word “bromance” and *Mian Ji* have been broadened beyond the limited description of gay relationships and now refers to friends and netizen gatherings, *Mian Ji* is an important part of gay culture. *Mian* here refers to communication happening in the physical space instead of the intermediary and represents the conversion of online communication to offline relationships.

**Figure 1:**
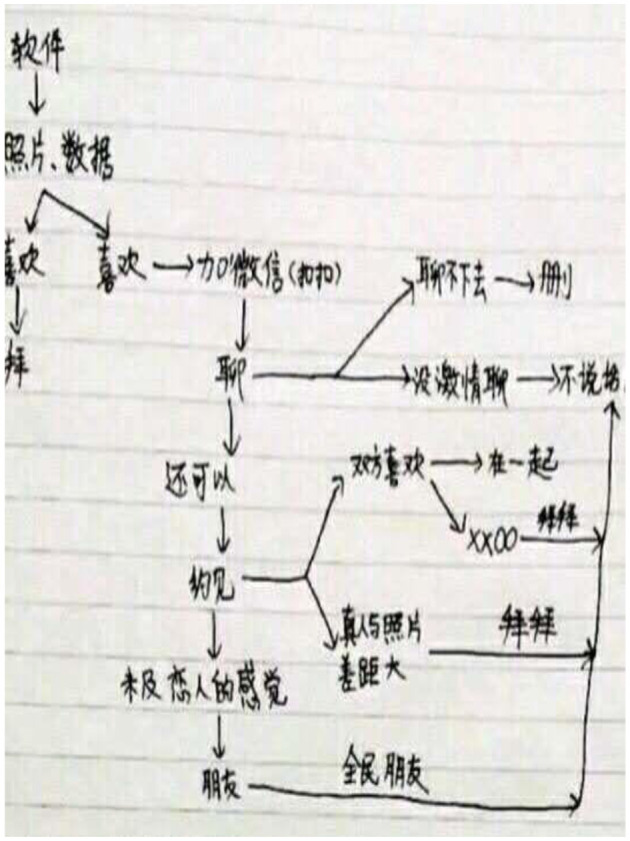
Process from Blued-based online chat to Mian Ji.

**Figure 2:**
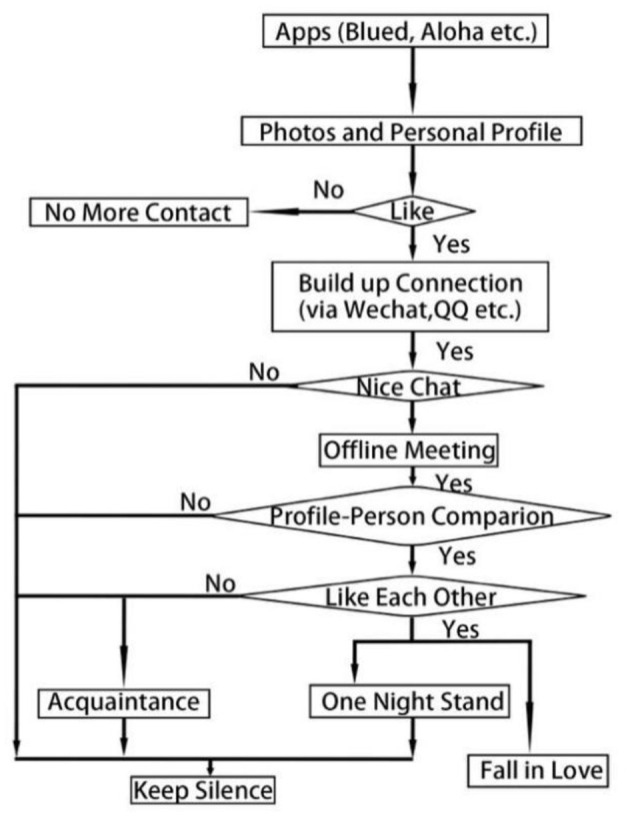
Process from Blued-based online chat to *Mian Ji* (in English).

On Blued, the transition from online interaction to offline generally requires a process, and this process is surprisingly consistent for most interviewees. Some users drew a flow chart presenting the process from “online chat on Blued” to *Mian Ji* (as shown in Figures 1, 2), including steps such as opening the application, browsing lists of people nearby (which are generally filtered based on the user's preferences), and looking at photos and information (such as age, height, weight, etc.). If no real photos are seen, a user may ask for photos. The conversation may stop if one user finds the other user's appearance or information unsatisfying. The user may add another user's QQ or WeChat *o*r maintain contact on Blued. If one of the users feels that the conversation is lacking, he may delete the other user or just end the conversation. For users who find a conversation interesting, the next stage should be having an offline meeting and then falling in love or having a sexual encounter. Whether they will get into a closer relationship or be normal friends depends on impressions at this meeting. From asking for photos to chatting on WeChat to face-to-face, the visibility of Blued users gradually deepened. In this process, mediated visibility becomes embodied visibility. If the other party is dissatisfied with the content disclosed in one of the steps, the next step of the relationship may be put on hold.

G usually speaks to others actively: “for example, if somebody's profile is attractive, I will proactively speak to him, but only if I'm bored.” When choosing whether greet or reply to others, B values height and age, and G stated the following:

These are the two most important factors. If somebody's height and age are to my taste, I will chat with him openly. I like being with a taller partner; if they are shorter than me, I may not be interested. When responding to others, I will always return to say hello, unless something is particularly busy or there is no time to return, I will return next time I respond to whoever talks to me. I will also look through his information and judge whether he deserves to have conversation according to his age and height. In terms of interactions, I've had video calls, which is the most intimate behavior that I've done. For someone even more attractive, I might ask for a date.

The process of *Mianji* is the process of transferring online accessibility to offline accessibility. In this process the impressions formed by online visibility are checked against the impressions formed during the offline interaction. If the offline impression is consistent with the online or the offline impression is better than the online, it will facilitate a deeper engagement. Otherwise, it will force the engagement to end. B and his first boyfriend met through this form:

“I was the one who first took the initiative to say hello to him. Although he did not put a real photo on Blued, the information on his profile, such as height, weight, age, is quite in line with my standards. After talking a few sentences, we sent each other photos, read and burn kind of, and both of us feel good. After chatting a few times, we met offline for a meeting and I think it went on well. I often encounter people who send me some false photos and information, but he did not. The impression I got of him after the meeting was no different from that I got when we talked online. After that, we added WeChat, and after a while, We became lovers.”

Some users are specifically picking the kind of impression is better, but some users such as G think:

“As long as I don't feel very bad when chatting online, I can meet them offline. The relationship between us mainly depends on the impression after meeting If the impression is good, try to pursue, if the impression is average to do ordinary friends is also very good.”

However, many users do not establish a relationship through offline meetings. One important reason is that the impressions formed during online interactions collapse when encountering offline visibility because many Blued users manage their online impressions with the help of fake photos, beauty filters, etc. F once experienced this contrast between online and offline.

“Once I had a good conversation with a guy online, and he showed me his picture, I was satisfied. After that, I made an appointment with him to meet in front of the school gate. I went a little late, and he was already waiting for me at the entrance. I took a look from afar, and the impression gap between online and offline is quite big, so I slipped away. Although this is quite rude, I feel that this person is dishonest, and there is no need to continue the relationship.”

### The cost of social accessibility: asymmetrical visibility and risk

As a kind of self-presentation, the location information on Blued is an essential reference for users to judge one other's identity. This presentation and the interaction between users, interwoven with the “familiarity” brought about by the proximity of geographical location, allows users to match their online identity to their offline identity. This presentation has negative consequences for the gay community. Among these consequences, there is a kind of right inequality between seeing and being seen. This results in what Hatuka and Toch (2017) call “asymmetrical visibility.”

Many interviewees, particularly those who had not come out publicly, were reluctant to let people around them know their gay identity. However, these gay men still need to socialize with others. This interaction is also an important reason why many gay men use Blued, not only because they want to find other gay men but also they want to be found. Blued users will do a lot to reach this goal, particularly deciding which personal information to share. For example, a user may put high-quality profile photos and a particular name, making an effort to reach standardized height and weight. Paradoxically, users are also afraid of being discovered, especially by people who know them in real life. On Blued, “although you can see others, others can also see you.” When a user finds someone close to him, he always guesses who he is in the physical spaces. As D revealed:

If showing zero kilometers, it means that this person is quite close to me; because he's right next to me, so I'd like to know who he is. Since gay men all have something in common, if I find someone zero kilometers away from me, when I'm in places like the library, I would raise my head and look around. Maybe I was too bored.

The presentation of the self on Blued is an essential clue for guessing who the user is. The clues include a profile photo, height, weight, age, photos, and distance. In the field study, both the researchers and the interviewees had the experience of judging “who is he?” through users' self-presentations. For A, it was unpleasant to be recognized.

A has worked in the Students' Union at both a college level and university level, allowing him to become acquainted with many people. Since A is the director of the College's Learning Department of Students' Union, he organizes various competitions, making him a celebrity at his college. However, A's position in the Students' Union also confines him. Due to his worry of being recognized, he does everything carefully on Blued, ensuring not to upload accurate information. The account name is XX, age 24, 178 in height, and 68 kg in weight. His signature is “How it feels to like a person.” The rest of the information on his profile includes “constellation (Pisces),” “Figure (neat).” All of the above information is untrue except his age. A hides in this way, stating the following: “Nothing but the age on Blued is real. I used to set my weight at 200 and my height at 200 deliberately. Using this method, people can't guess who you are at all. Even if they suspect who you are, they do not have proof and cannot figure it out.” For family reasons, A took a year off in the fourth year and returned as a senior. He took a job in Hangzhou during his year off from school. At his company, A does not use Blued; furthermore, his fear of exposure prevents him from opening Blued or logging in. N also met people that think in this way, stating the following: “I did not want to be recognized in a space like a school through the means that we've mentioned. I still don't want to come out but want to see other users.”

In the face of these risks, Blued users always take various measures to disguise themselves from others. However, in the close range, because of mutual acquaintance and the fact that users will reveal some information intentionally or unintentionally in the process of chatting, some users are often recognized by people who know them in real life. For example, A has been recognized by others and has recognized others as well. A and Jiang (for the sake of convenience, a pseudonym is used in this article) met when they were freshmen in military training and had added one another on WeChat. Although the two men were from the same college, their interaction was rare because they were from different majors. It was not until the summer vacation of their sophomore year when the two competed together for the position of freshman class assistant that they started to have contact during their role as a class assistant when a new term began. Coincidentally, A downloaded Blued at that time and happened to “find” Jiang:

His profile photo on Blued was a man in military uniform, but he didn't show his head; it was covered by something. I remembered that there was a time when I was holding an event, I happened to meet him while he was participating in the game. The picture he used for the competition was the profile photo on Blued. I thought of him and recognized that it was him.

A is an Engineering major whose class is composed mainly of boys; they all live in the same dormitory compound made up of two buildings, with the distance between the two buildings at ~20 m. According to the distance shown on Blued, it is easy for these students to determine if they are from the same college. It is easy to be recognized by others if they are from the same college along with frequent social activities.

A had also had the experience of being recognized by others on Blued but did not know whom they were, making A depressed:

There was a guy who should also be our senior. I don't know who he is, but, based on that distance and other information, I'm sure he's from our college. He kept trying to guess who I was for a few days, and then I told him straight. Later, he responded, ‘It's been good to chat so far and we have to end it' and blocked me. I don't feel good; I hate that other people know exactly who I am while I don't know anything about them.

A similar case happened to C, who found an acquaintance on Blued. C had uninstalled Blued for a while in college because he was too busy with university communities and departments to consider using the application. C then downloaded Blued again when he was free and found a man one day at a distance of 0 km, causing him to examine his profile information more closely:

He didn't have an avatar on his account, but he took a picture of his leg with my bed in the picture's background! I'd had doubts about his sexuality before but wasn't sure, and he hadn't mentioned it to me. Since we can see the record of visitors on Blued, I saw that he had found me and blocked me. I might have been recognized by him since my ID on Blued is also the name of my official account on WeChat, which is often seen by others. (C)

In the above case, Jiang was a “familiar stranger” to A, and C's roommate was also a “familiar stranger” to C. On Blued, the ideal state is “all strangers”; everyone on Blued should be anonymous. However, the familiarity generated from proximity, coupled with the unintentional revelation of identity on Blued, interrupts the mutual anonymity. As in A's case, there is an asymmetry of visibility between seeing and being seen. This asymmetry is the cost of accessibility. As N says, “Without coming out publicly, if you still want to date gay friends, you still have to pay some price, right?”

## Conclusion and discussion

In China, the public space for homosexual interaction in the past existed either in the marginalized physical space in gay bars, bathhouses, etc., or in the digital community. However, the gay community's imagination is organized by Blued based on the location of the user. Gay individuals can project the physical location of their bodies into cyberspace on Blued. Through the mediation of this digital interface, a queering code/space is created through the hybrid of physical space and digital space in the form of a locative structure. This system overcomes the limitations of traditional Dianr by making it public in a visual form and transforming the physical space dominated by heterosexual norms—where hand-holding, hugging, and kissing between men and women are considered normal, but between men are deemed abnormal—into a hybrid space for homosexual interaction. With the help of spatial filtering, gay individuals in an area can “see”each other within this space and engage in related social activities. To some extent, the modern city has become a place where “*Dianr* is everywhere.”

In the Chinese context, this means of communication has particular significance for most gay individuals. Much of the gay community in China is in the deep closet. Despite a growing number of individuals are coming out, many are too afraid to do so. These individuals want to know people of similar sexual orientations close to them but experience difficulties in a physical space dominated by heterosexuality. Blued cuts off heterosexual interactions, allowing gay men with social needs to see one another within a region. As a result, many subjects in the field study were surprised when they first used Blued to find so many people of their sexual orientation within such proximity. The visibility and accessibility based on *Blued* refresh the users' sense of place, contributing to a local group belonging and having important significance for Blued users to integrate into the local gay men. Although the social communication based on Blued is a hybrid communication that combines online and offline, the online behavior of Blued users mostly has a clear offline direction. The convenience of realizing these online relationships offline is Blued's unique advantage. The interaction of this hybrid space is initially carried out online. If one user wants to have a further relationship with another online, the interaction will immediately be extended to the offline in the form of a meeting or sexual encounter. Furthermore, Blued allows gay men to develop close friendships, extending their social network beyond that of heterosexuals and allowing them to “help” and “support” one another. Most Blued users are anonymous, which means that visibility on the application is limited. However, it is this limited visibility that allows gay men close to one another to be visible while also maintaining a “comfortable distance” (i.e., to remain anonymous and not be recognized by others). However, the hybrid of physical space and online social cues creates “familiar strangers,” in which there exists asymmetry between the seeing and being seen.

These interactions refer to gay individuals' public life in the heterosexual-dominated urban space. From traditional *Dianr* to Blued-based queering code/spaces, gay individuals in cities have been pursuing rights in the urban community. This right is highlighted at the spatial level in how to create a public space for queer interaction in a heteronormativity physical space, and how to enter, meet, date, interact in this space. The visibility of Blued has changed the definition of traditional *Dianr* within the city and weakened its role in meetings between members of the gay community (especially in physical spaces such as public toilets, parks, bathhouses, etc.). Therefore, it may be argued that the queering code/space provided on Blued is connected with the construction of the gay community in the urban public space, as well as with the gay community's rights in urban public space in the Chinese mainland.

Lefebvre (1996) believes that the current urban space is deeply influenced by the logic of capital, which causes problems such as alienation and isolation of urban space and leads to problems such as deprivation of participation. In this process, differences are marginalized. This urban right consists of comfortable residence, participation in decision making, space using, etc. Inheriting Lefebvre's view of urban rights, McQuire (2016) believes that the connotation and realization path of residents' rights to cities have undergone fundamental changes because of the conditions of new technologies. According to McQuire (2016) geomedia has become an important medium for different groups to realize urban rights at present. As a geomedia, Blued is also related to the spatial rights of the gay community. The space rights of the gay community are manifested in many aspects. This paper explicitly discusses the rights of the gay community to see and be seen in the daily living space and to carry out social communication. Platform improvements can be enacted through adjustments to platform configurations, aimed at bolstering the social inclusivity of the gay male community. These enhancements may involve implementing strategies to alleviate inequalities and discrepancies in visibility on the platform, granting users increased autonomy over their visibility, and concurrently addressing the propagation of misinformation. Such measures could significantly contribute to facilitating the Chinese gay male community in attaining greater visibility and agency within urban environments.

However, this publicity and these spatial rights are limited, reflected in three aspects: firstly, the queering code/space is about the visibility between gay groups, relatively closed visibility that insulates its members from heterosexuality. The gay community still lacks a broad social identity in real life. Secondly, this visibility is intraregional and constrained by geospatial distance, which is different from the open public space of online forums. Thirdly, the visibility between homosexuals and within the region has a particular ambiguity level, which is manifested in the uncertainty of geographical location (only distance is shown, whereas the specific location is not clear) and the selective display of online information (false information can be disclosed). Despite these limitations, Blued breaks through the limitations of the original interaction of gay men in mainland China and opens up new possibilities for local community integration and interaction of gay men, reflecting the significance of digital media for the social interaction of marginalized urban groups.

The issues and findings of this paper help promote research on social interaction between members of the gay community and public life in the digital age and are meaningful in theory to the binary opposition between reality and virtuality, physics and digital, etc. In the choice of research objects, this paper focuses on university students and urban white-collar workers who have just entered the job market, which has age and class limitations. For example, issues such as how middle-aged and elderly gay men use Blued, how people with limited education use Blued, the kinds of social interactions and group identities that these groups have established through Blued require further discussion.

## Data availability statement

The original contributions presented in the study are included in the article/supplementary material, further inquiries can be directed to the corresponding author.

## Ethics statement

The studies involving humans were approved by Guangdong Ocean University and Fudan University. The studies were conducted in accordance with the local legislation and institutional requirements. The participants provided their written informed consent to participate in this study. Written informed consent was obtained from the individual(s) for the publication of any potentially identifiable images or data included in this article.

## Author contributions

TX: Conceptualization, Data curation, Investigation, Methodology, Writing—original draft, Writing—review & editing. YZ: Data curation, Formal analysis, Investigation, Writing—review & editing.

## Footnotes

^1^For example, Fu's (2012) study highlights that eye contact is a vital physical cue for gay men to recognize and connect with each other in public spaces, facilitating interactions and conversations. In our research, interviewee D mentioned, “Gay men often exhibit certain characteristics, such as wearing makeup, having specific gestures, and displaying feminine behaviors, which typically suggest they are gay. At this point, I will intentionally look at him, and if he responds with eye contact, it's like we've established a connection, commonly known as ‘gaydar.' Following this, there might be a deliberate effort to start a conversation or interact.”

^2^QQ is an instant messaging software comparable to WeChat. Douban and Baidu Tieba are online communities centered around various interests. Each of these platforms includes group social features, which many gay men use to form and join communities for social interaction.

^3^ https://www.octoplusmedia.com/china-key-media-blued/

## Appendix

**Table A1:** Interviewee information.

**A**	**Student (senior)**	**Zhanjiang**	**Male**	**In-depth Interview**	**2019/4/29**
**B**	**Student (junior year)**	**Zhanjiang**	**Male**	**In-depth Interview**	**2019/5/8**
C	Student (senior)	Dongguan	Male	Voice Call on WeChat	2019/5/18
D	Student (junior year)	Zhanjiang	Male	In-depth Interview	2018/6/13
E	Student (junior year)	Zhanjiang	Male	In-depth Interview	2018/11/11
F	Student (junior year)	Zhanjiang	Male	WeChat Message	2018/11/22
G	Urban White-collar Workers	Shanghai	Male	In-depth Interview	2018/12/04
H	Urban White-collar Workers	Shanghai	Male	In-depth Interview	2019/01/05
I	Urban White-collar Workers	Shanghai	Male	In-depth Interview	2019/01/23
J	Student (Year 3 of High School)	Shanghai	Male	In-depth Interview	2019/01/27
K	Urban White-collar Workers	Shanghai	Male	In-depth Interview	2019/01/29
L	Student (Doctorate)	Shanghai	Male	Voice Call on WeChat	2019/02/22
M	Student (senior)	Zhangjiang	Male	In-depth Interview	2023/4/3
N	Urban White-collar Workers	Beijing	Male	In-depth Interview	2023/7/12
